# Evaluation of medical services from the perspective of COVID-19 vaccine demand satisfaction in Hangzhou, China

**DOI:** 10.3389/fpubh.2022.862283

**Published:** 2022-11-09

**Authors:** Mingjun Cheng, Yunchen Zhu, Peili Cen, Shan Huang

**Affiliations:** ^1^The Architectural Design and Research Institute of Zhejiang University Co., Ltd., Hangzhou, China; ^2^Center for Balance Architecture, Zhejiang University, Hangzhou, China; ^3^2nd Affiliated Hospital, School of Medicine, Zhejiang University, Hangzhou, China

**Keywords:** COVID-19, theory of planned behavior (TPB), population raster, vaccination sites, Hangzhou city

## Abstract

The outbreak of COVID-19 has had a huge global impact, and it continues to test the resilience of medical services to emergencies worldwide. In the current post-epidemic era, vaccination has become a highly effective strategy to prevent the spread of COVID-19. However, using conventional mathematical models to evaluate the spatial distribution of medical resources, including vaccination, ignore people's behaviors and choices and make simplifications to the real world. In this study, we use an enhanced model based on the Theory of People Behavior (TPB) to perform a macro analysis of the satisfaction ability of medical resources for vaccination in Hangzhou, China, and attribute the city to a three-level structure. According to the allocation, the supply capacity of vaccination sites is calculated and divided into four categories (good, normal, not bad, and bad). Meanwhile, we raise an assumption based on the result and the general development law of the city and analyze the reasons for the impact of personal behavior on the spatial distribution of medical resources, as well as the relationship between the demand distribution and spatial distribution of medical resources and future development strategies. It is considered that the overall medical resources, especially vaccination in Hangzhou, feature the situation of central supply overflow, and are found to hardly meet the needs of population points in surrounding areas, requiring a more flexible strategy to allocate facilities in these areas.

## Introduction

“Healthy city” is a concept that was proposed by the World Health Organization (WHO) in 1984. In the past 40 years, researchers have derived various healthy city planning goals and built corresponding evaluation systems. In China, the relevant research increased rapidly between 2009 and 2019 (1). Since 2019, COVID-19 has severely threatened all citizens' health systems, even those in the most developed countries. Governments all over the world have been urged to introduce political decisions aimed at containing the pandemic. Large-scale non-pharmaceutical interventions were put in place, such as social distancing, school closures, the isolation of symptomatic people and their contacts, and generalized lockdowns in some severely affected areas (2–4) . The long-term statistical results show that the higher the population density (mainly in urban areas), the higher the probability of a COVID-19 outbreak (5, 6). Currently, under the influence of the COVID-19 pandemic, both the Chinese government and academics consider that building a more stable healthy city and the corresponding medical system requires urban planning to intersect with other disciplines, such as medicine or public health management (7–9). Judged from the current epidemic prevention achievements, the defense capability of China and even major cities of the world against COVID-19 is still incomplete (10, 11).

In the post-COVID-19 pandemic era, it is an important guarantee for people's normal life that comprehensive epidemic prevention work is performed (12, 13). Above all, the government updates the epidemic data in real time by establishing an information system, laying a strong foundation for a “dynamic clearing case” in China; meanwhile, for individuals, getting vaccinated is the most important part of epidemic prevention work. Thus far, China has implemented three rounds of vaccination (the first dose, the second dose, and the booster dose). During the execution of the vaccination program, it was found that vaccination is likely to be offered at existing community healthcare centers, and if these centers cannot meet the actual needs, a temporary vaccination site in different forms will be constructed nearby.

Since 1997, China has launched the community healthcare center service (14). By 2016, more than 98% of cities were able to provide such services. During the COVID-19 vaccination work, community healthcare centers were selected as key vaccination sites. However, in the process of mass vaccination in Hangzhou, we also found that there were many cases of temporary suspension of vaccination sites, the addition of new vaccination sites, or the resumption of suspension of vaccination sites. The continuous change of vaccination site information also reflects the lack of coordinated vaccination arrangements. Therefore, it is necessary to evaluate the overall medical service capacity of Hangzhou in terms of medical function, especially regarding vaccination services.

Previous research results provide some data on the links between travel time, patient type, patient choice, and hospital visits. Huff proposed using the gravity analysis method to judge the relationship between the supply and demand of urban facilities (15, 16). During the following 30 years, represented by Radke, the corresponding mathematical system was improved according to the resource allocation of medical facilities, and this was gradually developed into the two-step floating catchment area (2SFCA) method (17, 18).

The TPB theory originates from the choice model, which is one of the important theoretical foundations of social psychology. It can be traced back to Fishbein's theory of multi-attribute attitude, which later evolved into the theory of reasoned action (19, 20). In 1991, Ajzen further deepened the theory of rational behavior, and formally proposed the TPB (21). This theory explains the general decision-making process of individual behavior from the perspectives of information processing and expected value theory (22, 23).

The TPB has been used effectively in research in many behavioral fields, as it can significantly improve the interpretation and prediction of behavior. In 2006, the British National Institute of Health and Clinical Optimization conducted a detailed investigation on which several behavioral selection models should be used in certain environments, and TPB was considered to be a relatively effective strategy. At present, there are few research on medical facilities based on TPB (24), especially in China. Studies on TPB theory in the analysis of medical resource allocation are mainly based on questionnaire surveys and the analytic hierarchy process. Nie Jinghong used the TPB model to identify the most important factor affecting patients' choice of hospital and concluded that it was the distance to the hospital, with the second factor being the scale of the hospital (25). Furthermore, Shen Yue et al.'s research on the survey of patients in a small number of hospitals in Shanghai reached the following conclusions: (1) Health-seeking behavior is principally affected by the availability of cars or other motorized vehicles; and (2) when the disease tends to be serious, the patient will be less sensitive to distance. Last year, our team carried out the overall evaluation of medical supplementary services by combining 2SFCA and TPB to build a mathematical model based on Hangzhou general hospital and population data and reported the phenomenon of medical resource run (26).

## Methods and data

### Enhanced model based on TPB

TPB is a theory mainly focused on how people behave (21). According to the principle of TPB, people's behavior is decided by three aspects: attitude toward behavior (A), degree of perceived behavioral control (B), and subjective norm (C). We summarize this theory in a simple formula to evaluate the degree of how people would conduct their behavior (D).


(1) $$\begin{array}{l} D=A^{*}B^{*}C \end{array}$$

Specifically, attitude toward behavior (A) refers to the degree to which a person has a favorable or unfavorable evaluation or appraisal of certain behavior. Degree of perceived behavioral control (B) refers to the perceived ease or difficulty of performing that behavior and is assumed to reflect experience as well as anticipated impediments and obstacles. Subjective norm (C) means the perceived social pressure on one to perform or not to perform a certain behavior. In this research, as shown in Formula (2), we use *S* to represent A, which denotes the scale of the facility, and *d* to represent B, which denotes the distance between every two facilities.


(2) $$\begin{array}{l} D=S^{*}d^{*}C \end{array}$$

As TPB is an appropriate theory to explain people's behaviors, we build an enhanced model to predict how people will act in a city and how allocation satisfies the needs of individuals according to Formulas (3)–(5).


(3) $$\begin{array}{l} E_{ij}=\frac{K_{i-min}D_{ij}}{\sum_{j=1}^{n}{K_{i-min}D}_{ij}}=\frac{K_{i-min}S_{j}^{\beta }d_{ij}^{\lambda }C}{\sum_{j=1}^{n}{K_{i-min}S}_{j}^{\beta }d_{ij}^{\lambda }C} \end{array}$$

where:

*i* denotes the different population points;

*j* denotes the different medical facility (hospital);

*K*_*i*−*min*_ denotes the prerequisite attitude of a certain kind of people;

*D*_*ij*_ denotes the willingness to travel to a hospital in the region;

$S_{j}^{\beta }$ denotes the scale of the facility at *j* and β is the influence coefficient of the facility scale;

$d_{ij}^{\lambda }$ denotes the distance between *i* and *j*, and λ denotes the influence coefficient of distance;

*E*_*ij*_ denotes the intention probability of a certain kind of people at *i* going to *j* to use the supplementary service.

In addition, prerequisite attitudes are preconditions that determine whether an action occurs. For an individual, when P=1, the behavior is possible; meanwhile, when P=0, the behavior is impossible. For the same kind of people, the *P*-values of different individuals in a crowd are not the same, but their preferences are relatively similar. Therefore, in a large-scale study, the weighted average of the *P*-values of different populations can be regarded as *K*_*i*−*min*_, and their value should be between 0 and 1, which shows the preference of this kind of people for this type of behavior. For example, if only people over 18 years of age can be vaccinated in a specific region, the *K*_*i*−*min*_ of people under this age limit in this region will be 0. Therefore, the *K*_*i*−*min*_ in that region can be replaced by the actual action proportion of the group in a certain period; in this article, we consider that *K*_*i*−*min*_ = 1, because everyone is assumed to be vaccinated in China.

The scale difference between general hospitals is obvious for patients, and the subjective willingness of patients to use a medical service is positively correlated with the scale of the general hospital. Thus, we consider that β = 1, as described previously. At the same time, the scale difference between vaccination sites is not obvious for patients; therefore, the impact of the scale of vaccination sites on the patient's willingness is considered to be the same, that is, β = 0.

In this study, we consider that the distance patients are willing to travel to seek medical treatment at the hospital is negatively correlated with perceived behavioral control. Through the trial and fitting of different values, we found that using λ = −2 based on our previous study is reasonable because patients have a stronger willingness to go to a large-scale hospital when they have a serious disease. However, patients are more sensitive to distance when they need daily medical services, so λ = −3 is more reasonable under this condition. Here, the distance is calculated by using the road network.

Once we have evaluated the intention probability of a person's behavior, assuming that there are *i* residential points and *j* supplementary service points in total, by calculating the preference of each residential point for different supplementary service points, the demand for medical resources of each residential point for a certain supplementary service point under the preference of choice can be obtained and is expressed as:


(4) $$\begin{array}{l} S_{j}^{{}^{\prime }}=\sum P_{i}\times E_{ij} \end{array}$$

In this formula, $S_{j}^{{}^{\prime }}$ represents the number of medical needs demanded by the hospital, *P*_*i*_ represents the total population of the region, and *E*_*ij*_ is the preference for the supplementary service in the region. After summation, we can obtain all of the demanded medical needs for supplementary services favored by the residents. Using Formula (4), the number of needs required by each supplementary service to satisfy the residents' wishes based on their choice can be obtained and then compared with the existing supplementary services.

Through comparison, we generated a difference between the supply sizes that are demanded and the supply sizes that can be supplied. This difference leads to a mismatch between the actual supply of available medical resources and the true demand of residents, which in turn leads to the inability of residents to access the desired amount of medical resources. Thus, we calculated the number of true resources that can be allocated to a certain point by taking the true demand of residents as the benchmark. The relevant expression is as follows:


(5) $$A_{i}Q=\;\frac{\sum {}^{\frac{P_{i}\times \;E_{ij}\times \;S_{j}}{S_{j}^{\prime }}}}{P_{i}}$$

where *A*_*i*_*Q* represents the accessibility of population points calculated by behavioral analysis from the perspective of demand to supply.

Next, we used this method to infer the total amount of medical resources available to individuals in different areas of Hangzhou under the TPB-generated results caused by individual willingness selection.

### Study area and data

**Figure 1:**
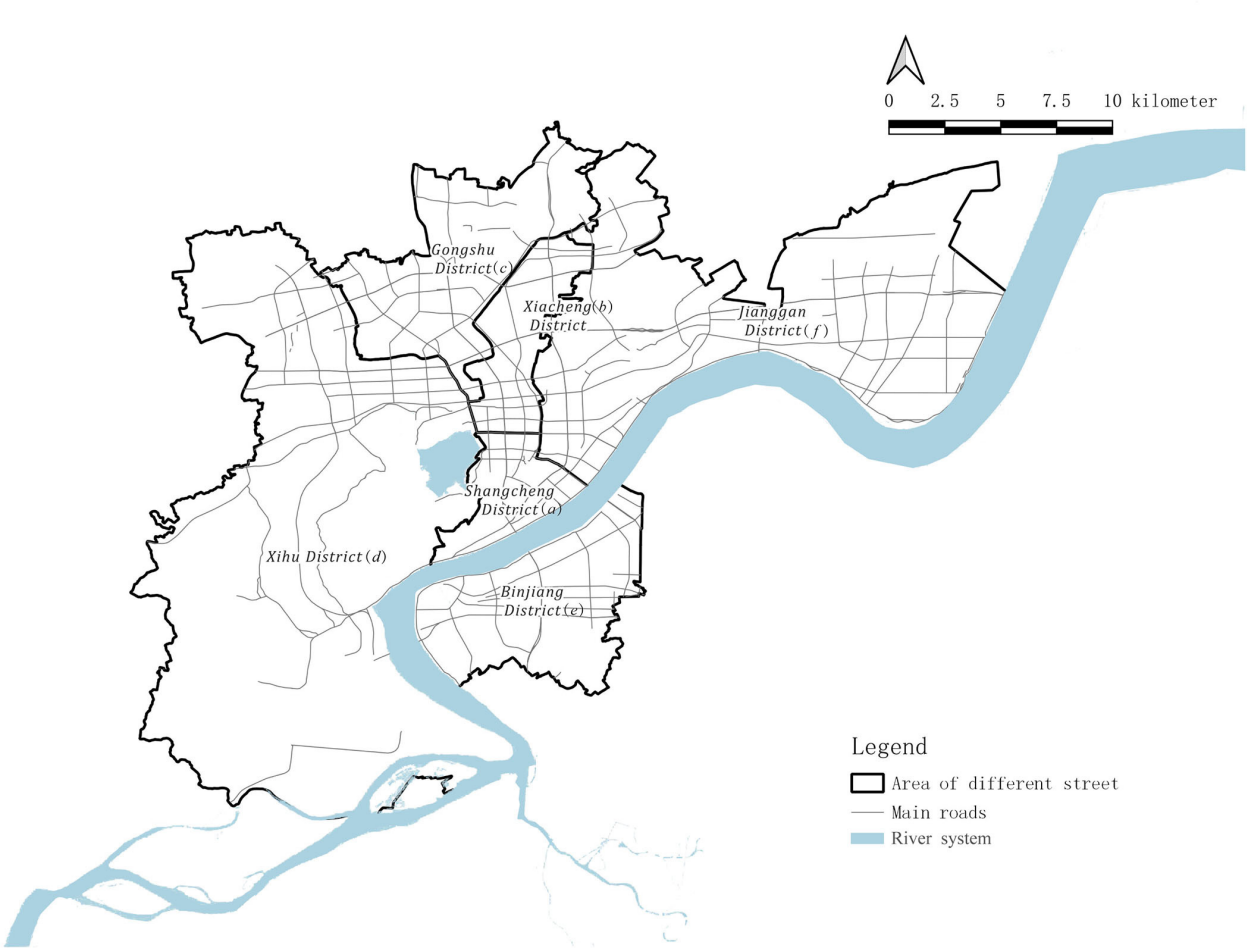
Basic information of six districts in Hangzhou city (road network, river system).

Hangzhou, as one of the central cities of the Yangtze River Delta, is the capital of Zhejiang Province and also its economic, political, and cultural center. The city has three famous World Heritage Sites, including West Lake, The Great Canal, and Liangzhu District, and its history has greatly shaped the present state of the city. This study considers the “main city area,” including six districts based on the “Hangzhou Urban Medical and Health Facilities Layout Plan (2016–2035, approved by the Hangzhou Government)” as the research area, including Shangcheng District (District a), Xiacheng District (District b), Gongshu District (District c), Xihu District (District d), Binjiang District (District e), and Jianggan District (District f). The subjects of this study include all residents in this area and vaccination sites at different levels (Figure 1).

It is important to note that not only do we focus on the traditional treatment that heals the disease but also on the comprehensive treatment needs, such as physical examination, medical care, pre-treatment, and emergency response to public health events. Therefore, this article considers that, in the COVID-19 pandemic era, the vaccination sites that provide vaccination and COVID testing for the entire society should well-represent the public vaccination sites in the area.

**Figure 2:**
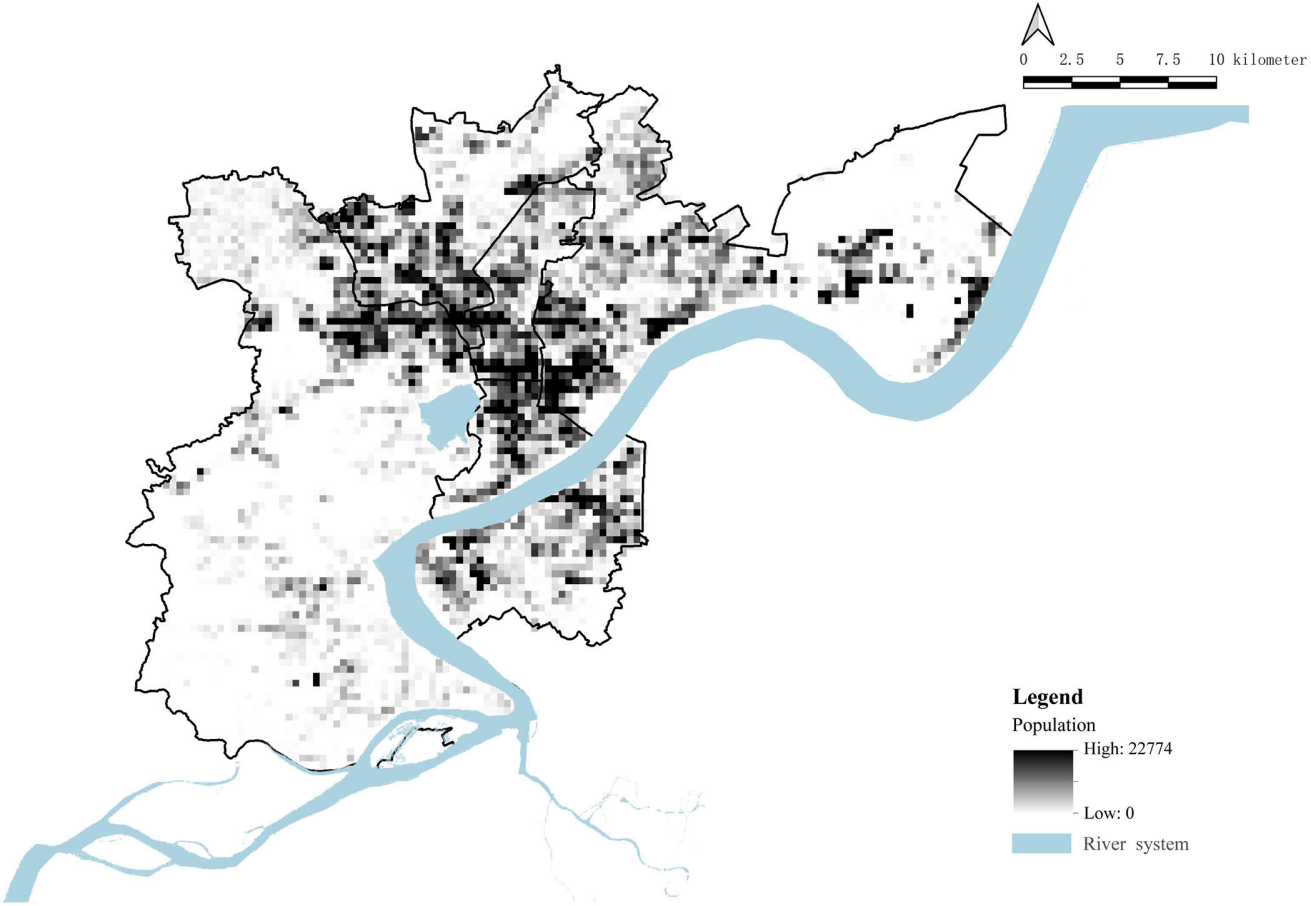
Population data of the six districts.

Before discussing the results, the following assumptions are made for both of the above aspects: This study takes the public medical resource provider as the service provider, including 68 vaccination sites with public information; the population in relation to the population demand point is that of the six districts of the main city of Hangzhou. We divide this population according to a 300 m × 300 m grid to obtain a total of 7,152 population grids, with a total population of 5.57 million (Figure 2) as the research object. The sensitivity of patients from population sites to the factor of distance to different levels of medical facilities is inconsistent. However, although the degree of people's intention to choose vaccination sites at various distances is generally different, it should not be assumed that vaccination sites that are farther than their medical address will not be chosen. Therefore, demand can be summarized as: “as long as the disease can be treated, people will go to any vaccination site in the six districts of the main city of Hangzhou.”

With respect to the different levels of vaccination sites, patients' needs are not the same. In general, since the service capacity of comprehensive vaccination sites is easy to perceive, the scale of such vaccination sites has an obvious impact on the patient's willingness to seek medical treatment. For comprehensive vaccination sites, the level of medical resources is often characterized by the number of beds. Therefore, we use this parameter to characterize the level of medical resources. At the same time, according to the requirements set forth by the “National Medical and Health Service System Planning Outline (2015–2020)” issued by the State Council in 2015, the number of beds in health institutions in China should reach 6 per 1,000 permanent residents by 2020. We use this item as an important indicator to evaluate whether Hangzhou's healthcare resources meet the basic needs of residents. This study is based on the data from vaccination sites in Hangzhou as of November 2020 (i.e., until the second dose is administered).

## Results

### Current situation and policy requirements for medical resources in Hangzhou

Herein, we consider six districts as the main city region based on the “Hangzhou Urban Medical and Health Facilities Layout Plan (2016–2035, approved by the government in July 2019)” as the research region, which includes Districts a, b, c, d, e, and f. All residents of the main city region are included in this work. Thus, we take general hospitals and vaccination points in Hangzhou as the two typical types of medical resources.

### Supply capacity of medical resources in Hangzhou

With regard to the integral distribution of vaccination points, the supply capacity of medical resources in terms of vaccination is approximately high in the west and low in the east of Hangzhou. The medical resource supply capacity of Districts a, b, c, and d (except Sandun Street) can easily meet or even exceed the medical needs of residents in these regions when compared with the local medical demand capacity. However, in Districts e, f, and Sandun Street in District d, the supply capacity of medical resources cannot satisfactorily meet the medical demands of residents.

**Figure 3:**
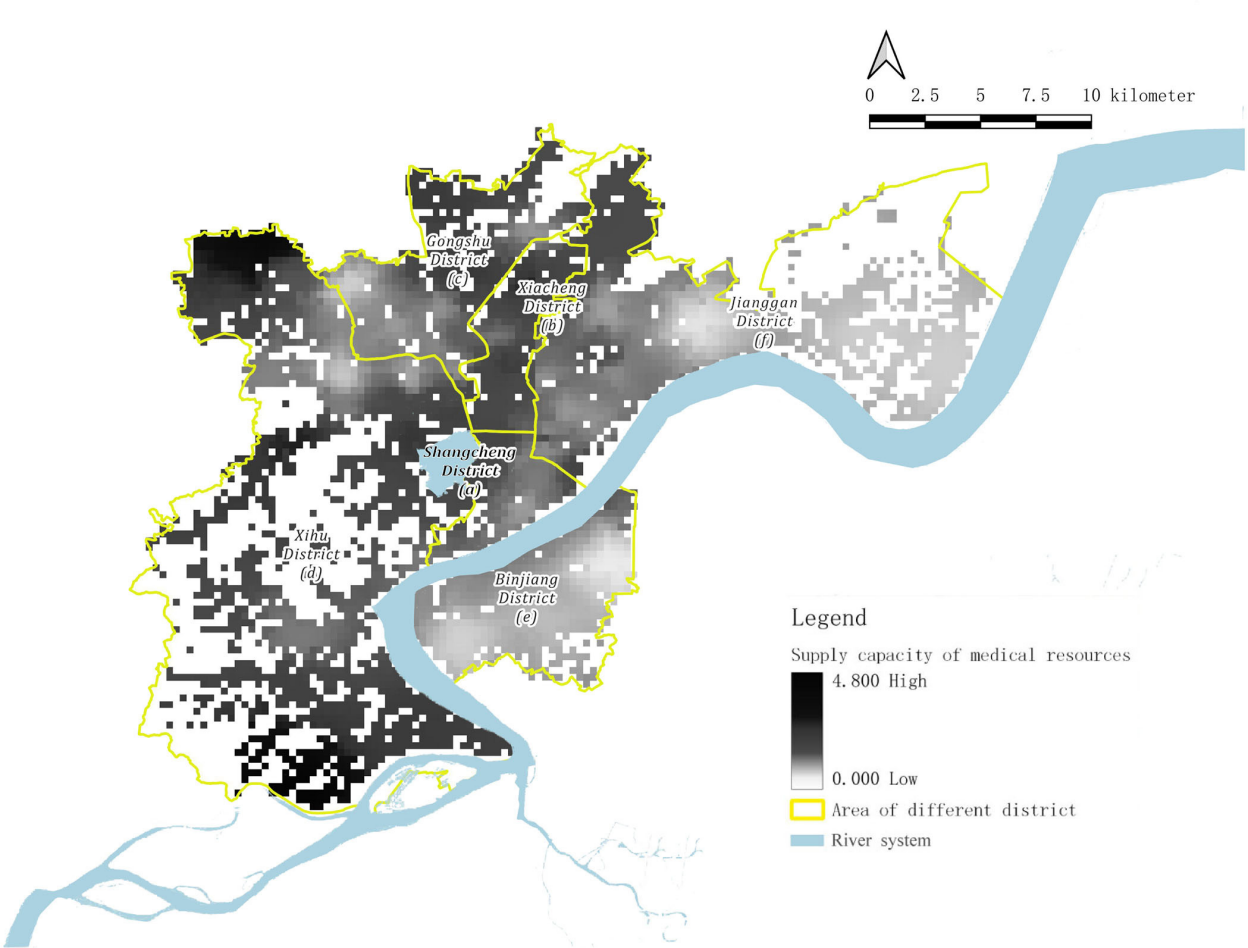
Analysis chart of supply capacity of medical resources in six districts.

As for the spatial distribution of vaccination sites, it can be divided into three types: overmet regions, unmet regions, and balanced regions (Figure 3).

The overmet regions include the Sandun Town area in the northwest corner, the Zhuantang area on the south side, the Kangqiao Street area on the north side, and the surrounding area of Zhejiang University City College, in which there is a higher basic medical service capacity.

The unmet regions include the whole area of District e, Wenxin Street, Zhaohui Street, Hemu Street, Xiangfu Street, Shiqiao Street, Ziyang Street, Jiubao Street in the west of the city, and the entire Xiasha area.

The balanced regions mainly include the areas that do not belong to the former two types, such as Xiaoying Street in District a, Changqing Street, Wenhui Street in District b, Shangtang Street in District c, Jianqiao Street, and Caihe Street in District f.

The detailed distribution of vaccination points in Hangzhou exhibits centripetal concentration and centrifugal dispersion. The vaccination points are concentrated in the north of District d, and Districts a, b, and c, which have experienced relatively long-time construction and development. Opposite to these areas, the distribution of vaccination points in Districts e and f are relatively scattered because of the comparatively short-term development in these areas.

**Figure 4:**
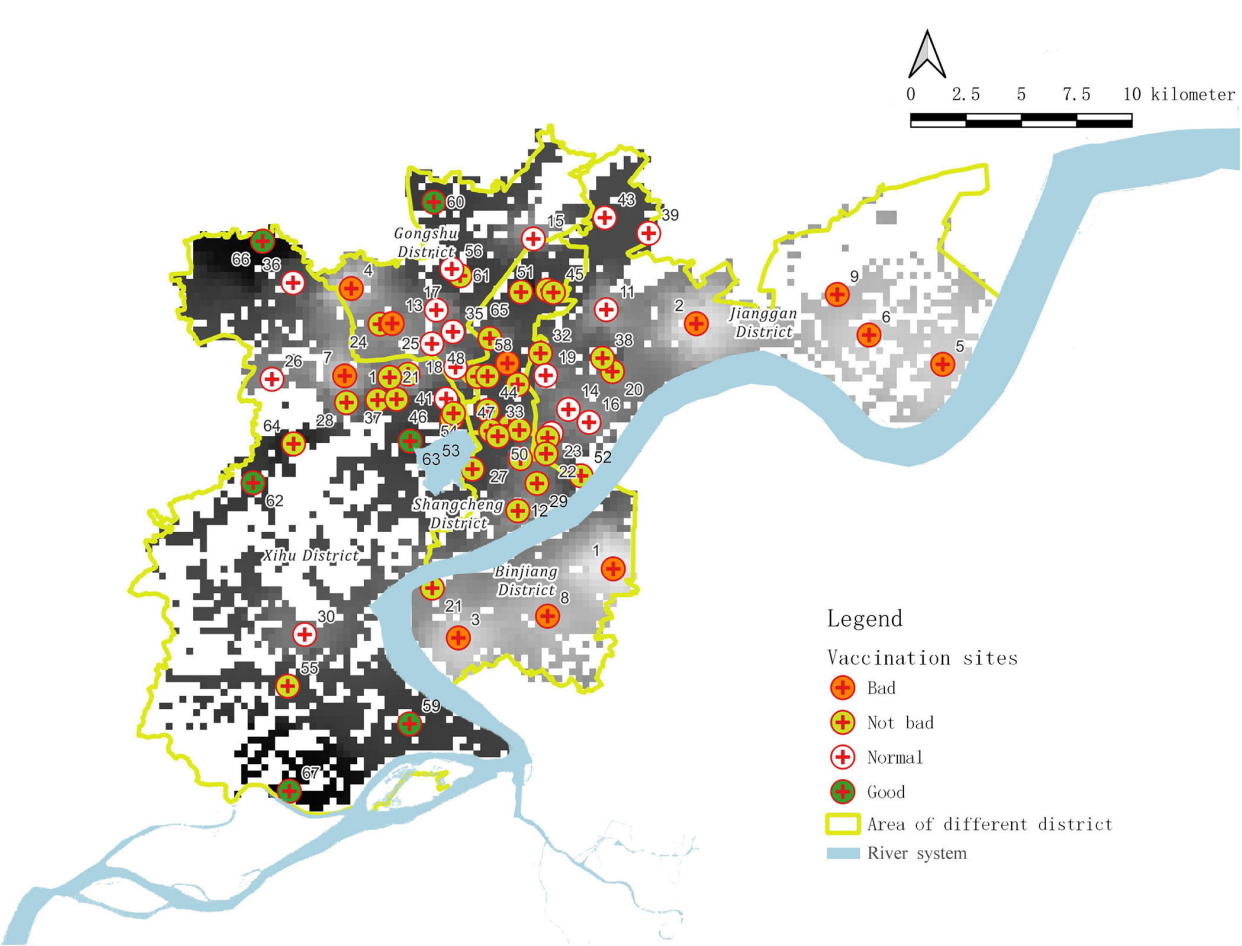
Analysis chart of medical supply capacity of vaccination sites.

Based on our analysis, the current medical resource points for vaccination in Hangzhou (67 points in total) can be subdivided into the following four categories (Figure 4, Table 1): bad, not bad, normal, and good.

**Table 1:** Evaluation of medical supply capacity for 67 vaccination sites.

**ID**	**Address**	**District**	**Total supply capacity (Formula 2)**	**Classification**
1	Yuanpu Community Health Service Center	Xihu district	47391.03	Good
2	Kangqiao Street Community Health Service Center	Gongshu district	46956.45	Good
3	Liuxia Street Community Health Service Center	Xihu district	44276.46	Good
4	Lingyin Street Community Health Service Center	Xihu district	43781.97	Good
5	Sandun Town makeshift vaccination site	Xihu district	24256.47	Good
6	Zhoupu Street Community Health Service Center	Xihu District	20831.15	Good
7	Hangzhou Jianggan district people's Jianqiao old hospital	Shangcheng district	102235.23	Normal
8	Kaixuan Street Community Health Service Center	Shangcheng district	95959.61	Normal
9	Banshan Street Community Health Service Center	Gongshu district	95764.81	Normal
10	Sijiqing Street Community Health Service Center	Shangcheng district	93955.78	Normal
11	Gongchenqiao Street Community Health Service Center	Gongshu district	93138.54	Normal
12	Mishixiang Street Community Health Service Center	Gongshu district	92271.40	Normal
13	903 Hospital of PLA joint logistics support force	Shangcheng district	92145.76	Normal
14	Zhejiang Youth Hospital	Shangcheng district	88795.11	Normal
15	Xiaohehushu Street Community Health Service Center	Gongshu district	88387.88	Normal
16	Jiangcun Street Community Health Service Center	Xihu district	86440.41	Normal
17	Zhuantang Street Community Health Service Center	Xihu district	82277.53	Normal
18	Daguanshangtang Street Community Health Service Center (Daguan)	Gongshu district	72086.51	Normal
19	Sandun town Community Health Service Center	Xihu district	69362.40	Normal
20	Dinglan Street Community Health Service Center	Shangcheng district	66416.56	Normal
21	Xixi Street Community Health Service Center	Xihu district	65570.48	Normal
22	Dingqiao District, Hangzhou Hospital of Traditional Chinese Medicine	Shangcheng district	64176.11	Normal
23	Daguanshangtang Street Community Health Service Center (Shangtang)	Gongshu district	51875.27	Normal
24	Cuiyuan Street Community Health Service Center	Xihu district	104980.81	Not bad
25	Ziyang Street makeshift vaccination site	Shangcheng district	99170.80	Not bad
26	Pengbu Street Community Health Service Center	Shangcheng district	90898.27	Not bad
27	Binjiang district makeshift vaccination site	Binjiang district	90633.77	Not bad
28	Caihe Street makeshift vaccination site	Shangcheng district	90480.97	Not bad
29	Xiangfu makeshift vaccination site	Gongshu district	88400.48	Not bad
30	Xiaoying Street makeshift vaccination site	Shangcheng district	85378.11	Not bad
31	Gudang Street Community Health Service Center, temporary vaccination site	Xihu district	83587.50	Not bad
32	Wangjiang Street makeshift vaccination site	Shangcheng district	83193.57	Not bad
33	Dongxing Street Community Health Service Center	Xiacheng district	81015.01	Not bad
34	Zhalongkou Street Community Health Service Center	Shangcheng district	76949.20	Not bad
35	Chaoming Street makeshift vaccination site	Xiacheng district	73699.38	Not bad
36	Changqingchaoming Street Community Health Service Center	Xiacheng district	72167.15	Not bad
37	Gudang Street Community Health Service Center	Xihu district	67293.97	Not bad
38	Pengbu Street Hailiangxin-Huixing hospital makeshift vaccination site	Shangcheng district	66979.48	Not bad
39	Caihe Street Community Health Service Center	Shangcheng district	66163.18	Not bad
40	Wenhui Street Community Health Service Center	Xiacheng district	64982.91	Not bad
41	Tianshuiwulin Street community Health Service Center	Xiacheng district	62985.40	Not bad
42	Shiqiao Street makeshift vaccination site	Xiacheng district	62684.27	Not bad
43	Cuiyuan Street makeshift vaccination site	Xihu district	61504.63	Not bad
44	Wulin Street makeshift vaccination site	Xiacheng district	59751.63	Not bad
45	Zhaohui Street Community Health Service Center	Xiacheng district	59150.54	Not bad
46	Zhaohui Street makeshift vaccination site	Xiacheng district	58435.12	Not bad
47	Hubin Street makeshift vaccination site	Shangcheng district	58320.98	Not bad
48	Shiqiao Street Community Health Service Center	Xiacheng district	56432.31	Not bad
49	Sijiqing Street makeshift vaccination site	Shangcheng district	56384.94	Not bad
50	Qingbo Street makeshift vaccination site	Shangcheng district	56379.82	Not bad
51	Beishan Street makeshift vaccination site	Xihu district	53079.74	Not bad
52	Zhuantang Street makeshift vaccination site	Xihu district	52972.26	Not bad
53	Beishan Street Community Health Service Center	Xihu district	51200.10	Not bad
54	Zhejiang University City College makeshift vaccination site	Gongshu district	45063.41	Not bad
55	Mobile temporary vaccination position of Wanxiang vocational and Technical College	Xihu district	36290.63	Not bad
56	Dongxin Street makeshift vaccination site	Xiacheng district	30000.36	Not bad
57	Xixing Street Community Health Service Center	Binjiang district	193439.36	Bad
58	Jiubao Street makeshift vaccination site	Shangcheng district	177005.54	Bad
59	Puyan Street Community Health Service Center	Binjiang district	146446.25	Bad
60	Xiangfu Street Community Health Service Center	Gongshu district	143798.26	Bad
61	Baiyang Street Wenchao Community Health Service Center	Jianggan district	143676.36	Bad
62	Baiyang Street Community Health Service Center	Jianggan district	143236.37	Bad
63	Wenxin Street Community Health Service Center	Xihu district	134105.83	Bad
64	Changhe Street Community Health Service Center	Binjiang district	123171.84	Bad
65	Xiasha Street Community Health Service Center	Jianggan district	119466.85	Bad
66	Hemu Street Community Health Service Center	Gongshu district	97952.26	Bad
67	Wenhui Street makeshift vaccination site	Xiacheng district	48133.48	Bad

From Table 1, we can find that 11 vaccination points qualify as “bad,” including Xixing Street Community Health Service Center, Jiubao Street Temporary Inoculation Point for Fangcang, and Puyan Street Community Health Service Center, accounting for 16.42%. These points (community health and medical service centers) cannot meet the vaccination needs of the surrounding areas, and no additional emergency vaccination points are provided nearby. At the same time, additional unreasonable emergency vaccination points are included.

Moreover, 33 vaccination points were evaluated as “not bad,” including Cuiyuan Street Community Health Service Center, Ziyang Street Temporary Vaccination Point, and Pengbu Street Community Health Service Center, accounting for 49.25%. These points (community health and medical service centers) cannot meet the vaccination needs of the surrounding areas, but the addition of emergency vaccination points nearby helps to meet the requirements.

The “normal” category includes 17 vaccination points, including Kaixuan Street Community Health Service Center, Banshan Street Community Health Service Center, and Sijiqing Street Community Health Service Center, accounting for 25.37%. These points (community health and medical service centers) can meet the vaccination needs of the surrounding areas, but they cannot meet the vaccination needs of wider areas.

Only six vaccination points are evaluated as “good,” including Yuanpu Community Health Service Center, Kangqiao Street Community Health Service Center, and Liuxia Street Community Health Service Center, accounting for 8.96%. These points (community health and medical service centers) can meet the vaccination needs of surrounding areas, and at the same time, they can take into account the vaccination needs of wider areas.

## Discussion

Previously, traditional and common methods like 2SFCA (17, 18), Huff models (15, 16), and others (27–31) were used to analyze the reasonableness of the allocation of facilities and the means to improve it. Meanwhile, few scholars have been concerned about the actual behavior of people in a city, let alone building a model to explain and predict it (32). At the same time, due to the rising demand for both the quality and quantity of medical services in China, it is becoming increasingly important to influence residents' medical behavioral preferences, which phenomenon has led to overcrowded large hospitals. The existing methods lack the mathematical expression of people's subjective demands and behaviors. In addition, traditional methods mainly contain two implicit conditions: All residents have the same demands and preferences; All residents choose a nearby facility, that is, they perform no-choice behavior, which simplifies the actual world. During the past 2 years, some researchers have attempted to analyze the basic medical services in the post-pandemic era, but resorted to using traditional methods (33–35). In contrast, we use the enhanced TPB model to improve the analytical power of predicting how people would choose a facility.

According to the enhanced TPB model, the regions of Hangzhou are categorized into three types (overmet, unmet, and balanced), and their descriptions are given as follows:

Overmet regions: These are mainly characterized by a lower population, which leads to lower demand for and moderate density of medical facilities. Therefore, each medical facility can easily meet the medical demand within a certain geographical range.

Unmet regions: Among them, areas with low medical resources can be further divided into three types as follows:

The whole area of Binjiang District (District e) and Jiubao Street. This is a new construction area of the city with a relatively fast-growing population and sizeable administrative area, which leads to the slow construction of basic medical facilities amid the high speed of urban construction and development. Therefore, the density of basic medical facility service points is relatively low, which causes the inferior medical service capacity of basic public resources in this region.

Wenxin Street, Zhaohui Street, Hemu Street, Xiangfu Street, Shiqiao Street, Ziyang Street, and so on, in the western part of the city. This area is an urban construction area that has already been completed. The population density and the total population are both relatively high, and the demand for the quantity and quality of basic medical facilities is also relatively high. However, in these unmet areas, the number and distribution density of medical facilities cannot keep up with the population's demand, resulting in the poor medical service capacity of basic public resources in these areas. Taking Wenxin Street as an example, the population density is relatively high, and the vaccination capacity is in short supply. Despite this condition, no new temporary vaccination point has yet been established, leading to the low efficiency of the actual vaccination process in this area.

Residents of certain streets are aware of the lack of basic medical facilities in their areas. From the results above, taking Xiangfu Street as an example, when it was realized that the area's vaccine service capacity was in short supply, a temporary vaccination site was set up in the south of this street. However, from a cross-street perspective, this is very close to the health service of Hemu Street, which caused an excess of medical resource supply. Also, this measure is not helping to effectively relieve the shortage of medical service supply for vaccination in Xiangfu Street. Therefore, we suggest that the resource distribution vaccination network needs to be comprehensively arranged on a large scale to avoid similar situations.

Xiasha area. This part of the wider region is extensive, and most of it is occupied by universities. A university always builds its basic medical facilities to serve itself, which increases the basic public resource service capacity of this region. However, it is still insufficient.

Balanced regions, where medical resources can be just satisfied, are mainly those that do not belong to the former two types of regions. Here, the residents' autonomous choice behaviors will form the “invisible hand” of the market for the allocation of medical resources. The medical resources available to residents in these areas are in relatively close proximity.

We found that Hangzhou has an apparent three-level structure of “the old town, the new city and the rural areas.” Old town refers to the old urban area where the construction and development have been completed and possesses the most resources. The new city means that the area is still developing at high speed and keen to have more facilities to meet the needs. And the rural areas refer to the rural parts that have not yet been planned for urban development and construction. Interestingly, the medical resource supply capacity of rural areas is usually not at the worst level; in fact, it is relatively satisfactory in many cases. This is because the rural population is comparatively small, such that these areas can meet the overall medical needs without large-scale or high-density medical facilities.

In addition to comparing the different areas, we also evaluated the facilities. The four main categories listed in Table 1 correspond to four kinds of supply capability of a medical resource (bad, not bad, normal, and good).

Limited by history, “bad” vaccination sites are mainly located in the city areas that have stopped in their development progress. The scale of these facilities is too small to handle public health emergencies, and thus, they cannot be used as strong medical service providers most of the time. Therefore, these facilities must cooperate with general hospitals and even other venues to ensure the satisfaction of medical needs (such as streets in the Shangcheng District (District a), in which the original community service centers fail to meet the needs of vaccination, and it must add temporary facility points instead of using the original community service center). If these areas cooperated with some general hospitals, they could be regarded as “not bad” vaccination sites.

Since “normal” and “good” vaccination sites have more adequate venues, more even distribution, and more sufficient medical resources, these sites can be used as effective providers of basic medical resources and can meet the emergency medical needs in the corresponding part of the city. This category of vaccination sites mainly includes recently constructed street health service centers and vaccination sites converted from some private hospitals, among others.

There are specific situations where several kinds of homogeneous populations are concentrated in specific regions (mainly universities), and a designated vaccination site needs to be set up in these regions to meet the needs of these people. These vaccination sites are not necessarily covered by public healthcare networks because they are special supplements to the city healthcare networks. Another possible situation is that, if an area is difficult-to-enter and there is a medical facility in its area, that facility can easily form an access barrier for public resources at the area boundary to ensure that residents in the area are given priority services.

To explain the above results, we raise the following assumption. Based on the general law of urban development, a small number of hospitals could meet the healthcare needs well before the city's growth and population explosion. At this stage, only a few small healthcare centers, such as drug stores, rather than healthcare facilities and very few large hospitals are needed, especially in suburban areas. However, during the past 30 years, the city's growth and population explosion made old facilities too small to cover all needs in a growing city area. Moreover, the market rules leading to the lag in the construction of healthcare facilities render basic public healthcare facilities insufficient in the long term, placing this area in the unmet category (such as the west of the city). When an area stops growing (usually the oldest part of the city), which means that market selection and balance have been achieved, the constructed facilities can meet the increased healthcare needs, and the medical resource supply is relatively moderate, making it a balanced area. It is suggested that if some areas (mostly suburban areas, such as Jiubao, Xiasha, and Binjiang area) do not have a significant population that is expected to increase rapidly, new facilities should be built in advance to avoid turning into an unmet area, even though this place would become an overmet area in the short term. In brief, we believe that the result of the current allocation is caused by both historical reasons, like an undeveloped economy, and city planning that is not predictable enough to catch the growth rate of the city.

According to the results and our assumptions, we believe that being flexible in the location, construction, and total volume of medical facilities is the most important factor in dealing with the pandemic of COVID-19. Besides, in this post-pandemic era, the ability to handle a pandemic quickly is the key to preventing it. Although large comprehensive hospitals are more efficient in providing medical care, such facilities cannot be constructed in short periods such as 7 days, which is the critical threshold to prevent the virus from spreading. Under the existing conditions, a large number of facilities are still needed in a flexible way to handle emergencies during the pandemic in Hangzhou, China.

## Conclusion

In this work, we investigated COVID-19 vaccine demand satisfaction by evaluating the level of medical services in Hangzhou, China. We used the enhanced TPB model to replace the traditional method that ignores individuals' opinions and behavior. We also put forward the subjective concept of “vaccination attitude P” in the evaluation of medical vaccination services in Hangzhou, China, which needs to be accomplished through sample surveys or other methods in other countries. Based on the results of the enhanced TPB model, the overall situation of medical resources in Hangzhou is that the supply in the city center is overmet, while it is difficult to satisfy the needs of populations in the surrounding areas, requiring the medical resources to be more flexible. The suggested approach to increasing the flexibility includes the construction of new hospitals, setting up branches, building community healthcare centers, and establishing a systematic, multi-level, and moderate-distance distribution medical and health space network. The current study still has the following limitation: certain included vaccination points are community health service centers without vaccination services on several campuses, which cannot be accounted for at present.

## Data availability statement

The original contributions presented in the study are included in the article/supplementary material, further inquiries can be directed to the corresponding author/s.

## Ethics statement

Ethics approval and written informed consent were not required for this study in accordance with national guidelines and local legislation.

## Author contributions

All authors listed have made a substantial, direct, and intellectual contribution to the work and approved it for publication.

## Funding

This work was supported by the construction and scientific research projects in 2021 of Center for Balance Architecture, Zhejiang University (Award number: K-20212791).

## Conflict of interest

Author MC and YZ were employed by the Architecture Design and Research Institute of Zhejiang University. The remaining authors declare that the research was conducted in the absence of any commercial or financial relationships that could be construed as a potential conflict of interest.

## Publisher's note

All claims expressed in this article are solely those of the authors and do not necessarily represent those of their affiliated organizations, or those of the publisher, the editors and the reviewers. Any product that may be evaluated in this article, or claim that may be made by its manufacturer, is not guaranteed or endorsed by the publisher.
